# Effect of Surface Treatment of Halloysite Nanotubes (HNTs) on the Kinetics of Epoxy Resin Cure with Amines

**DOI:** 10.3390/polym12040930

**Published:** 2020-04-17

**Authors:** Vahideh Akbari, Maryam Jouyandeh, Seyed Mohammad Reza Paran, Mohammad Reza Ganjali, Hossein Abdollahi, Henri Vahabi, Zahed Ahmadi, Krzysztof Formela, Amin Esmaeili, Ahmad Mohaddespour, Sajjad Habibzadeh, Mohammad Reza Saeb

**Affiliations:** 1 Department of Resin and Additives, Institute for Color Science and Technology, Tehran P.O. Box: 16765-654, Iran; vahidehakbari1991@gmail.com; 2 Center of Excellence in Electrochemistry, School of Chemistry, College of Science, University of Tehran, Tehran 11155-4563, Iran; maryam.jouyandeh@gmail.com (M.J.); smrparan@gmail.com (S.M.R.P.); ganjali@ut.ac.ir (M.R.G.); 3 Biosensor Research Center, Endocrinology and Metabolism Molecular-Cellular Sciences Institute, Tehran University of Medical Sciences, Tehran 11155-4563, Iran; 4 Department of Polymer Engineering, Faculty of Engineering, Urmia University, Urmia 5756151818-165, Iran; h.abdollahi@urmia.ac.ir; 5 Université de Lorraine, CentraleSupélec, LMOPS, F-57000 Metz, France; 6 Department of Chemistry, Amirkabir University of Technology, Tehran 1591634311, Iran; zahmadi@aut.ac.ir; 7 Department of Polymer Technology, Faculty of Chemistry, Gdańsk University of Technology, Gabriela Narutowicza 11/12, 80–233 Gdańsk, Poland; krzysztof.formela@pg.edu.pl; 8 Department of Chemical Engineering, School of Engineering Technology and Industrial Trades, College of the North Atlantic–Qatar, 24449 Arab League St, Doha 24449, Qatar; amin.esmaeili@cna-qatar.edu.qa; 9 Department of Chemical Engineering, College of Engineering and Technology, American University of Middle East, Egaila 15453, Kuwait; ahmad.mohaddespour@mail.mcgill.ca; 10 Department of Chemical Engineering, Amirkabir University of Technology (Tehran Polytechnic), Tehran 1591634311, Iran; sajjad.habibzadeh@aut.ac.ir

**Keywords:** cure kinetics, halloysite nanotubes, epoxy, isoconversional method

## Abstract

The epoxy/clay nanocomposites have been extensively considered over years because of their low cost and excellent performance. Halloysite nanotubes (HNTs) are unique 1D natural nanofillers with a hollow tubular shape and high aspect ratio. To tackle poor dispersion of the pristine halloysite (P-HNT) in the epoxy matrix, alkali surface-treated HNT (A-HNT) and epoxy silane functionalized HNT (F-HNT) were developed and cured with epoxy resin. Nonisothermal differential scanning calorimetry (DSC) analyses were performed on epoxy nanocomposites containing 0.1 wt.% of P-HNT, A-HNT, and F-HNT. Quantitative analysis of the cure kinetics of epoxy/amine system made by isoconversional Kissinger–Akahira–Sunose (*KAS*) and *Friedman* methods made possible calculation of the activation energy (*E*_α_) as a function of conversion (α). The activation energy gradually increased by increasing α due to the diffusion-control mechanism. However, the average value of *E*_α_ for nanocomposites was lower comparably, suggesting autocatalytic curing mechanism. Detailed assessment revealed that autocatalytic reaction degree, *m* increased at low heating rate from 0.107 for neat epoxy/amine system to 0.908 and 0.24 for epoxy/P-HNT and epoxy/A-HNT nanocomposites, respectively, whereas epoxy/F-HNT system had *m* value of 0.072 as a signature of dominance of non-catalytic reactions. At high heating rates, a similar behavior but not that significant was observed due to the accelerated gelation in the system. In fact, by the introduction of nanotubes the mobility of curing moieties decreased resulting in some deviation of experimental cure rate values from the predicted values obtained using *KAS* and *Friedman* methods.

## 1. Introduction

The curing reaction of epoxy resin, so-called crosslinking, includes formation of short chains followed by branching until formation of a cross-linked network [1,2,3]. Curing process of epoxy progresses further by the occurrence of gelation and vitrification transitions that affect the ultimate properties of the cured system [4,5,6]. The cure reaction of epoxy and consequently its properties are additionally dependent on the fillers involved in the formulation and the interactions between filler and the matrix [7,8,9]. The interfacial interaction between fillers and polymer matrix can be improved by adjusting the surface chemistry of the reinforcing agents. Moreover, the morphology of nanoparticles and their content can strongly affect cross-linking reaction of epoxy. The cure study of epoxy containing 0D spherical cobalt doped Fe_3_O_4_ nanoparticles revealed that the amount of heat of cure of nanocomposite was significantly higher than that for the neat epoxy system due to the catalytic effect of nanoparticles [10]. In another study, surface modification of cobalt doped Fe_3_O_4_ nanoparticles with ethylenediaminetetraacetic acid enhanced the curability of epoxy nanocomposite because of the reaction between the carboxylic acid anchored to the surface of particles and the epoxide rings [11]. Nonisothermal differential scanning calorimetry (DSC) results indicated that epoxy nanocomposite containing Ni-Al-NO_3_ 2D layered double hydroxide platelet-like nanoparticles increased the cross-link density of network compared to the unfilled epoxy due to the reaction of nitrate anion with epoxide ring [12]. In a recent study, it was found that introduction of 0.1 wt.% microporous 3D metal–organic framework into the epoxy significantly improved the heat release by 63% [13]. 

Among 1D nano-scale fillers, halloysite nanotubes (HNTs) are of interest of researchers because of being inexpensive and having a highly reactive surface [14,15,16]. In recent years, many studies published on the effect of HNT as filler on the thermal stability, mechanical, anticorrosion, and flame retardant properties of polymers [17,18,19,20,21,22]. Biocompatibility and availability are two important factors placing reason behind selection of HNTs [23,24]. However, there is still a shortage of resources in the study of network formation and the effects of thermal phenomena on the properties of HNT/epoxy nanocomposites. Vahedi et al. [25] studied isothermal cure behavior of epoxy/HNT nanocomposites with two different curing agents of diaminodiphenylmethane (MDA) and diethylenetriamine (DETA). They indicated that the curing behavior of the cured epoxy with DETA was not severely affected by the addition of HNTs. However, HNTs showed catalytic effect on the curing behavior of MDA cured epoxy due to the presence of hydroxyl groups on the surface of HNTs. The effect of 0.5, 1.0 and 2.0 wt.% of HNTs on the curing behavior of epoxy/anhydride systems was also discussed by Jouyandeh et al. [26]. The results indicated that the crosslinking reaction was promoted at 0.5 and 1.0 wt.% of HNTs, while the curing reaction was hindered at 2.0 wt.% of HNTs because of deactivation of anhydride curing agent fueled by diffusion into the lumen of HNTs.

In a recent work, pristine HNT (P-HNT) was modified by alkali activation process (A-HNT) and epoxy silane functionalization (F-HNT) as well. The potentials of P-HNT, A-HNT and F-HNT to cure with epoxy and amine were studied qualitatively by nonisothermal DSC [27]. In order to gain more information about cure reaction of epoxy containing P-HNT, A-HNT and F-HNT, *Cure Index* (*CI*) was used [28]. However, to unravel complexities in the system, e.g., the effect of each type of nanotubes on the autocatalytic and non-catalytic crosslinking reactions, we needed isoconversional kinetic analyses to provide more detailed quantitative information on crosslinking reactions by patterning the evolution of activation energy of systems as a function of conversion and the orders of the aforementioned crosslinking reactions. 

In this work, cure kinetics of epoxy/HNT nanocomposites was comprehensively studied through quantitative analysis of network formation in the epoxy/amine systems filled with P-HNT, A-HNT and F-HNT. Following the previous work, we more accurately investigate the curing reaction, accomplished by the calculations based on integral Kissinger–Akahira–Sunose (*KAS*) and differential *Friedman* isoconversional methods.

## 2. Materials and Methods

### 2.1. Materials

Bisphenol A diglycidyl ether (EPON™ Resin 828) epoxy resin with epoxide equivalent weight of 450–550 g/eq. was provided by Hexion (Shanghai, China). The curing agent of Epikure™ F205 with hydrogen equivalent weight (HEW) value of 105 g/eq. was provided by Hexion (Shanghai, China). HNTs with lumen diameter of 15–70 nm, outer diameter of 50–200 nm and the length of 100–3000 nm was purchased from Imerys Tableware Asia Limited (Auckland, New Zealand).

### 2.2. Preparation of Epoxy Nanocomposites

Pristine halloysite nanotubes (P-HNTs), alkali-activated HNTs (A-HNTs), and epoxy silane (2-(3,4-epoxycyclohexyl) ethyltriethoxy silane)-functionalized A-HNTs (F-HNTs) were prepared according to the previous work [19]. For preparation of epoxy/HNTs nanocomposites, 0.2 wt.% P-HNTs, A-HNTs and F-HNTs were separately added to the epoxy resin under sonication for 30 min. Then, the amine curing agent was added to the resulting nanocomposites at 2:1 resin:curing agent ratio at room temperature and stirred for 3 min. The prepared samples were stored at −4 °C prior to calorimetric measurements.

### 2.3. DSC Measurement

Cure reactions of epoxy in the presence of P-HNTs, A-HNTs and F-HNTs were studied by nonisothermal DSC on a model DSC1 Mettler device (Greifensee, Switzerland). Samples of about 15 mg were analyzed under nitrogen atmosphere at heating rates of 5, 10, 15, and 20 °C/min. The nonisothermal DSC test was performed in the temperature range between the room temperature and 300 °C to cover the whole curing process. 

## 3. Results and Discussion

**Figure 1:**
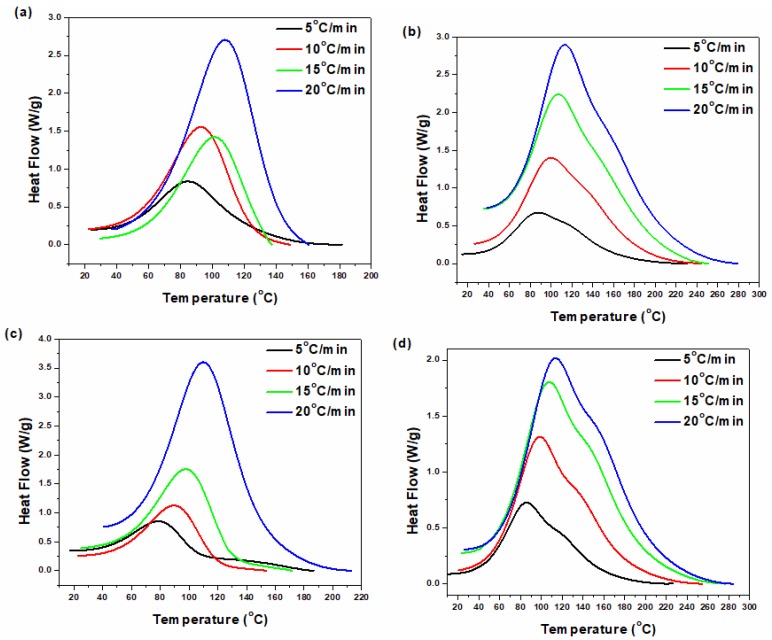
DSC thermographs of the (**a**) neat epoxy, (**b**) epoxy/P-HNT, (**c**) epoxy/A-HNT and (**d**) epoxy/F-HNT at four different heating rates of 5, 10, 15 and 20 °C min^−1^ [27].

Based on a comprehensive protocol recommended for the analysis of cure process in thermoset composites [29], the results of nonisothermal DSC at four heating rates of 5, 10, 15 and 20 °C/min were analyzed for neat epoxy and its nanocomposites. Figure 1 shows the DSC data of the neat epoxy and its nanocomposites including 0.2 wt.% of P-HNT, A-HNT and F-HNT. DSC thermograms for each sample were shifted towards higher temperatures by increasing the heating rate, suggesting enhanced kinetic energy of the system at higher heating rates [30,31,32]. Observation of a single exothermic peak in the thermogram of the neat epoxy confirmed the single-step curing kinetic assumption [33,34]. The existence of a small shoulder in the case of epoxy/P-HNT nanocomposite was the characteristics of the complexity of curing reaction in this system. In fact, the presence of hydroxyl groups on the surface of P-HNT with less reactivity compared to the amine groups of curing agent resulted in epoxide ring opening at the later stages of curing reaction. As a result, a small shoulder was observed in the DSC thermograms at high temperatures. In the case of A-HNT incorporated epoxy system, this shoulder was disappeared due to the alkali activation of HNT where some loci of the inner surface of HNT were etched and the OH groups decreased in number compared to the P-HNT [27]. However, OH groups were formed during the reaction of epoxy on the surface of F-HNT with amine groups of curing agent by surface modification of HNT with epoxy silane coupling agent, and again a shoulder appeared at higher temperatures. Therefore, P-HNT and A-HNT assisted curing agent, while F-HNT somewhat compensated the stoichiometry for epoxy resin. 

### 3.1. Cure Behavior

**Figure 2:**
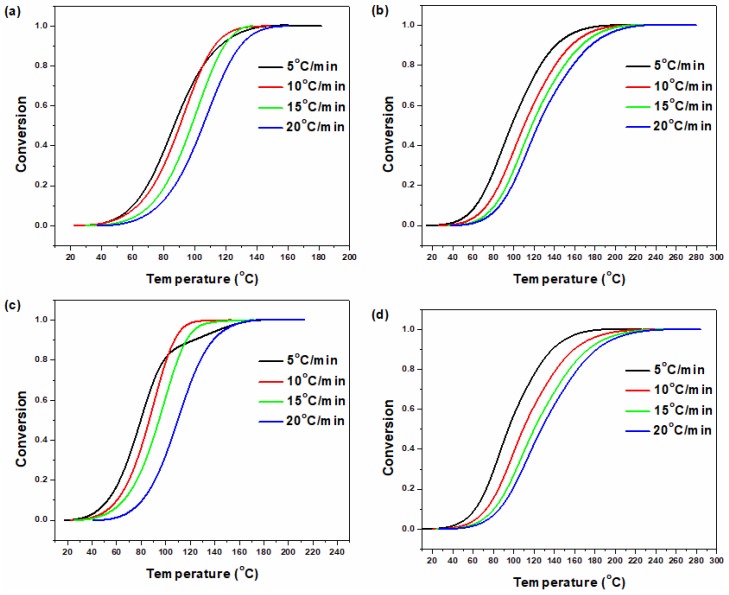
Fractional extent of conversion for neat epoxy and its nanocomposites at heating rates of (**a**) 5, (**b**) 10, (**c**) 15 and (**d**) 20 °C min^−1^.

Studying the cure behavior of epoxy nanocomposites helps one to collect information on the role of nanoparticles on accelerating or decelerating the cure reaction rate. Curing conversion (α) is one of the first evaluation terms for studying the cure behavior of thermosetting systems, derived from the Equation (1) [35]:

(1) $$\alpha =\frac{\Delta H_{T}}{\Delta H_{\infty }}$$

where Δ*H_T_* is the heat of cure at a certain temperature and Δ*H_∞_* is the total heat of cure reaction. The data calculated from Equation (1) for neat epoxy and its nanocomposites as a function of curing time are depicted in Figure 2. The sigmoidal shape of conversion curves observed for the neat epoxy, epoxy/P-HNT, epoxy/A-HNT and epoxy/F-HNT was served as a signature of the autocatalytic nature of curing process [36]. In the beginning of the curing reaction, *α* was increased slowly until reaching gel point where a dramatic increase in the extent of reaction was observed, and ultimately again the α increased slowly until the complete cure [37].

### 3.2. Cure Kinetics

The rate of epoxy cure reaction is measured by the following equation:

(2) $$\frac{d\alpha }{dt}=k(T)f(\alpha ),$$

In Equation (2), *f*(*α*) is the reaction model and *k(T)* is the reaction rate constant, which is defined based on Arrhenius equation as [38]:

(3) $$k(T)=Aexp\left(-\frac{E_{\alpha }}{RT}\right),$$

where *A* is the pre-exponential (also known as frequency factor), *R* is the universal gas constant and the *E_α_* is the activation energy of the curing reaction. 

By substituting Equation (3) into the Equation (2), the rate of cure can be obtained as:

(4) $$\frac{d\alpha }{dt}=Aexp\left(-\frac{E_{\alpha }}{RT}\right)f(\alpha ),$$

For nonisothermal curing reaction, Equation (4) can be reformed as follows by introducing the heating rate (*β* = *dT*/*dt*):

(5) $$\frac{d\alpha }{dT}=\left(\frac{A}{\beta }\right)exp\left(-\frac{E_{\alpha }}{RT}\right)f(\alpha ),$$

Model-free (isoconversional) method was used for evaluating kinetic parameters of epoxy system in the presence of P-HNT, A-HNT and F-HNT. According to the isoconversional models, the reaction rate at a given conversion (α) is merely a function of temperature [39]. The model-free isoconversional method is divided into two types of differential and integral methods. Among the differential isoconversional methods, the *Friedman* method is the most frequently used one, defined as [40]:

(6) $$ln{\left(\frac{d\alpha }{dt}\right)}_{\alpha }=ln\left[f(\alpha )A_{\alpha }\right]-\frac{E_{\alpha }}{RT_{\alpha }},$$

The activation energy is calculated from the slope of the plot of *ln(dα/dt)_α_* against *1/T_α_* at a certain *α*.

The *KAS* is a well-known accurate integral isoconversional method, which calculates the activation energy through the slope of the curve of $ln\left(\beta_{i}/T_{\alpha ,i}^{2}\right)$ against *1/T* by the following equation [41]:

(7) $$ln{\left(\frac{d\alpha }{dt}\right)}_{\alpha }=ln\left[f(\alpha )A_{\alpha }\right]-\frac{E_{\alpha }}{RT_{\alpha }},$$

**Figure 3:**
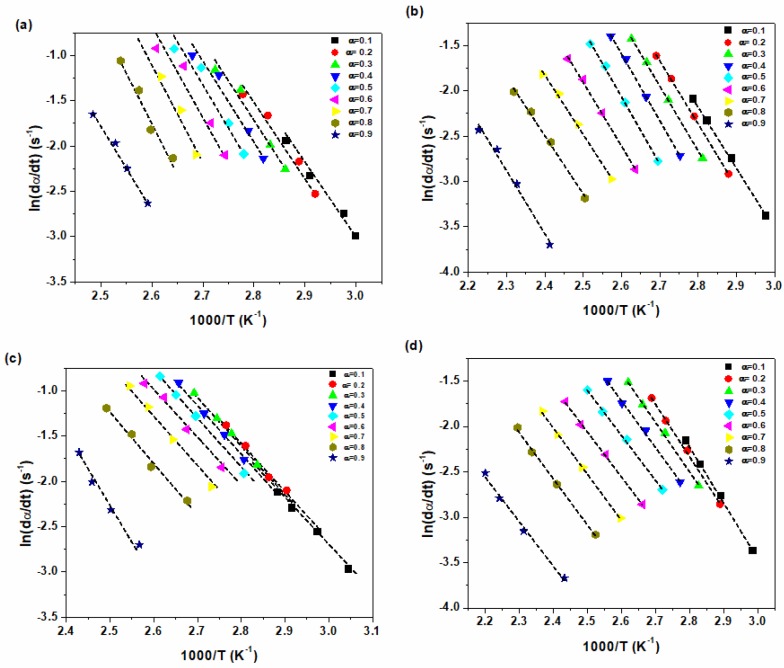
Plots of *ln(dα/dt)* vs. *1/T* for (**a**) epoxy resin, (**b**) epoxy/P-HNT and (**c**) epoxy/A-HNT and (**d**) epoxy/F-HNT nanocomposites based on *Friedman* model.

**Figure 4:**
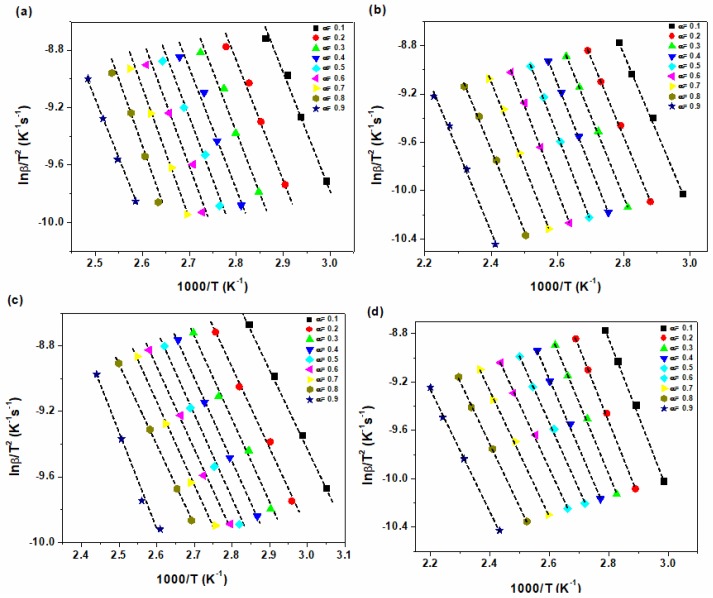
Plots of *ln(β/T^2^)* vs. *1/T* for (**a**) epoxy resin, (**b**) epoxy/P-HNT and (**c**) epoxy/A-HNT and (**d**) epoxy/F-HNT nanocomposites based on *KAS* model.

Figure 3 and Figure 4 show the typical isoconversional plots based on *Friedman* and *KAS* methods, respectively. The fitted lines in the *Friedman* and *KAS* methods are parallel with each other at 0.2 < α < 0.9, suggesting a single cure kinetics mechanism exists in this α range. This also indicates the inaccuracy of models at the initial and final stages of curing reaction when the cure processes is under the control of chemical reaction and diffusion, respectively [42]. 

**Figure 5:**
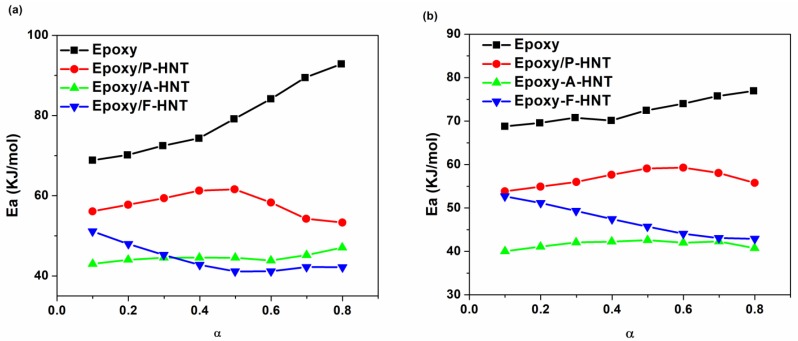
Variation of *E_α_* versus conversion for epoxy resin and prepared nanocomposites derived from (**a**) *Friedman* model and (**b**) *KAS* model.

**Figure 6:**
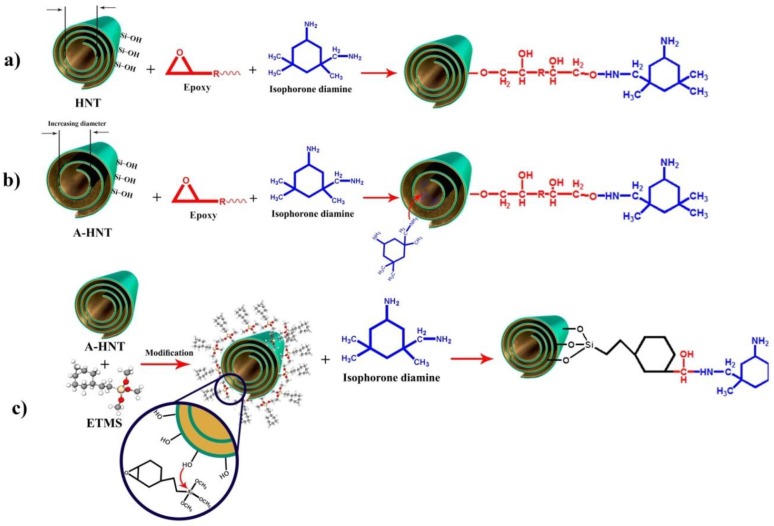
Possible reaction of (**a**) P-HNT and epoxy, (**b**) A-HNT with epoxy and amine curing agent and (**c**) F-HNT with amine group of curing agent.

Figure 5 shows the changes in activation energy of cure reaction versus the extent of reaction for the epoxy resin and its nanocomposites containing 0.2 wt.% P-HNT, A-HNT and F-HNT based on the *Friedman* and *KAS* models. Besides, for explicit specification, a schematic illustration of the epoxy resin and its nanocomposites are depicted in Figure 6.

As expected, the data from the *Friedman* and *KAS* models are relatively alike. It should be noted that the predicted activation energies in the range of α > 0.8 and α < 0.2 are generally unreliable. According to Figure 5, the activation energy values for the neat epoxy are higher than the nanocomposites. The activation energy for neat epoxy increases gradually by increasing the curing conversion due to the decrement of the free volume of the system as the crosslinking reactions occurs. As a result, the activation energy is increased in the late curing stage due to the gelation, glass transition and the rise in the reaction viscosity when the reaction is controlled by the diffusion [43]. 

As can be observed from Figure 5, the activation energy decreased for samples including nanofillers. Addition of P-HNT into the epoxy matrix results in growth of heat of cure and decline in activation energy due to the increase in the number of effective interactions caused by the presence of OH groups on the surface of HNT that facilitate the curing reaction [44]. Moreover, the activation energy is slowly increasing at lower conversion rates, due to the reduction in free volume fueled by the gelation phenomenon. By contrast, at higher conversions the hydroxyl groups of HNT can participate in the epoxide ring opening at late stage of curing [45]. Amine groups of curing agent are more reactive than the hydroxyl groups on the surface of P-HNT; therefore, they participate in epoxy ring opening in the early stage of cure through the interaction of primary and secondary amines of curing agent with the epoxy groups. In the presence of P-HNT with hydroxyl-rich surface, catalytic curing reaction takes place via etherification reaction when the amine groups of curing agent are consumed or somehow remained unreacted in the gelled network in the later stage of cure [46] (Figure 6a).

According to Figure 5, the activation energy values for samples containing active and modified HNTs (A-HNT and F-HNT) are less than the other two samples. Reduction in activation energy for the A-HNT/epoxy cannot principally be a signature of facilitated cure, instead springs from the lack of evolution in the curing reaction. Alkali activation of HNT results in removal of the inner surface of nanotube as Al(OH)_3_ sheets, which cause fall in the inner hydroxyl groups and rise in the inner diameter [27]. Therefore, in the epoxy/A-HNT system the hydroxyl groups decreased compared to P-HNT and consequently the possibility of reaction between OH groups and epoxy rings should be decreased. On the other hand, the amino groups that are responsible for the curing reaction are probably trapped inside the nanoparticles and become inaccessible, resulting in an incomplete curing reaction (Figure 6b). 

Conversely, by modification of surface of HNT by silane coupling agent the activation energy decreases beside the increases in heat of cure indicates facilitation of crosslinking reaction. Epoxy silane coupling agent on the surface of F-HNTs can react with amine groups of curing agent which prevents re-aggregation of nanotubes in epoxy/curing agent system and results in more stable dispersion. Moreover, hydroxyl groups form during the reaction of epoxy groups on the surface of F-HNT with amine groups of curing agent as shown in Figure 6c, so the etherification reaction could be more highlighted and the cure process push towards to the reaction between hydroxyl groups and epoxy ring.

#### 3.2.1. Determining Reaction Model

Determination of the reaction model is an important step for deeper understanding that whether addition of P-HNT, AHNT and F-HNT can change the curing reaction mechanism. So, the goal is to find appropriate *f*(*α*) which correlate as close as possible with the experimental data. For epoxy system the nth order or two component autocatalytic reaction models could be considered. The *n*th order reaction model is defined as follows:

(8) $$f(\alpha )=\;{\left(1-\alpha \right)}^{n},$$

Moreover, Sestak and Berggren [47] proposed an empirical kinetic model as follows:

(9) $$f(\alpha )=\alpha^{m}\;{\left(1-\alpha \right)}^{n},$$

where *m* and *n* are the reaction orders. Friedman and Malek methods are two common approaches for determination of reaction model.

##### Friedman Method

Based on the Friedman method, the curing reaction model for epoxy system in the presence of P-HNT, A-HNT and F-HNT can be determined using Equation (10).

(10) $$ln\left[Af(\alpha )\right]=ln\left(\frac{d\alpha }{dt}\right)+\frac{E}{RT}=lnA+nln\left(1-\alpha \right),$$

**Figure 7:**
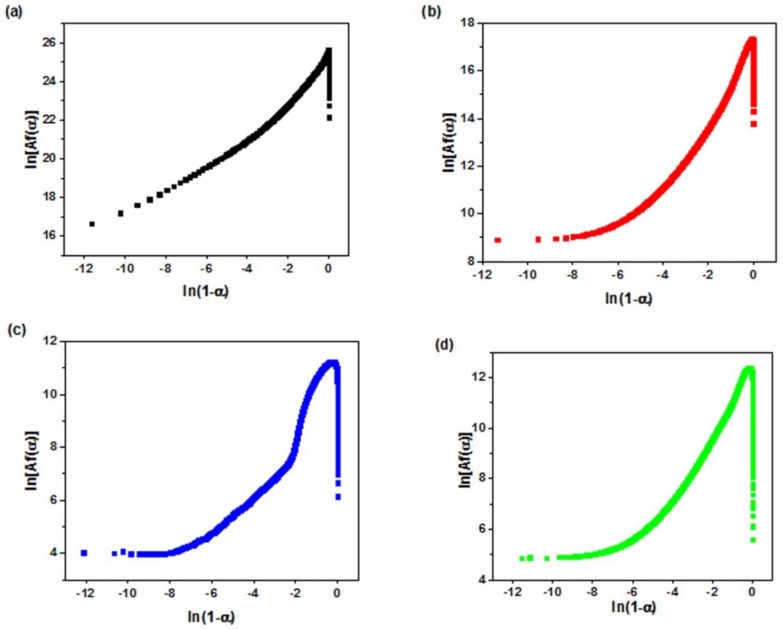
Plots of *ln [Af(α)]* vs. *ln(1 − α)* for (**a**) neat epoxy, (**b**) epoxy/P-HNT, (**c**) epoxy/A-HNT and (**d**) epoxy/F-HNT.

The plot of *ln[Af(α)]* as a function of *ln(1 − α)* for neat epoxy and its nanocomposites is shown in Figure 7 which shape denotes the deviation from nth order reaction. A straight line was obtained for noncatalytic nth order cure mechanism as a result of plotting *ln[Af(α)]* vs. *ln(1 − α)*. As it is clear in Figure 7, the *Friedman* curves for both neat epoxy and its nanocomposites show a maximum with in the conversion range between 0.2–0.4 which is indicative of autocatalytic reaction mechanism.

##### Malek Method

The kinetic model based on the Malek method can be determined using the following functions:

(11) $$y(\alpha )={\left(\frac{d\alpha }{dt}\right)}_{\alpha }exp\left(\frac{E_{0}}{RT_{\alpha }}\right)=Af(\alpha ),$$

(12) $$z(\alpha )={\left(\frac{d\alpha }{dt}\right)}_{\alpha }T_{\alpha }^{2}\left[\frac{\pi (x)}{\beta T_{\alpha }}\right],$$

The term in the bracket of Equation (12) can be omitted due to its low impact on the shape of the *z(α)* function. The constant *E*_0_ value in Equation (11) can be determined by *FWO* method (where the activation energy does not change with variation of *α* as follows:

(13) $$ln\;\left(\beta_{i}\right)=Const-1.052\;\left(\frac{E_{\alpha }}{RT_{\alpha }}\right),$$

**Figure 8:**
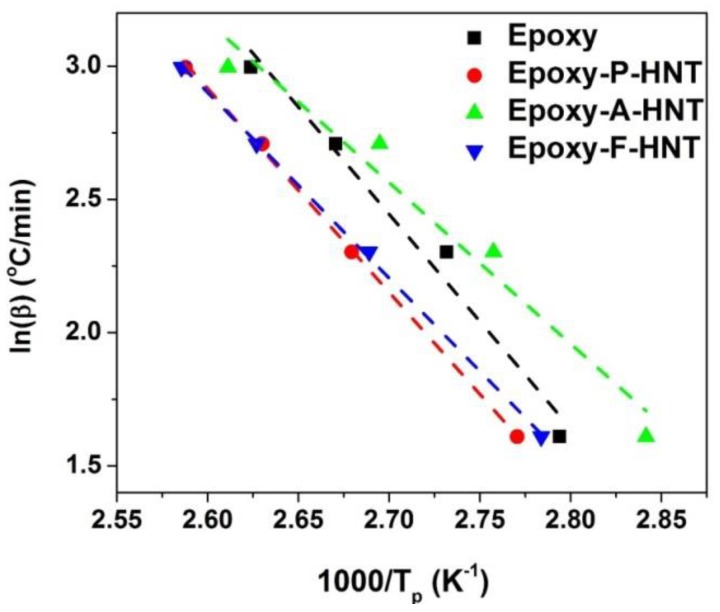
Plots of *ln(β_i_)* vs. *1/T* for neat epoxy, epoxy/P-HNT, epoxy/A-HNT and epoxy/F-HNT nanocomposites based on *FWO* model.

The activation energy is determined from the slope of *ln(β_i_)* vs. *1/T* as shown in Figure 8. 

**Figure 9:**
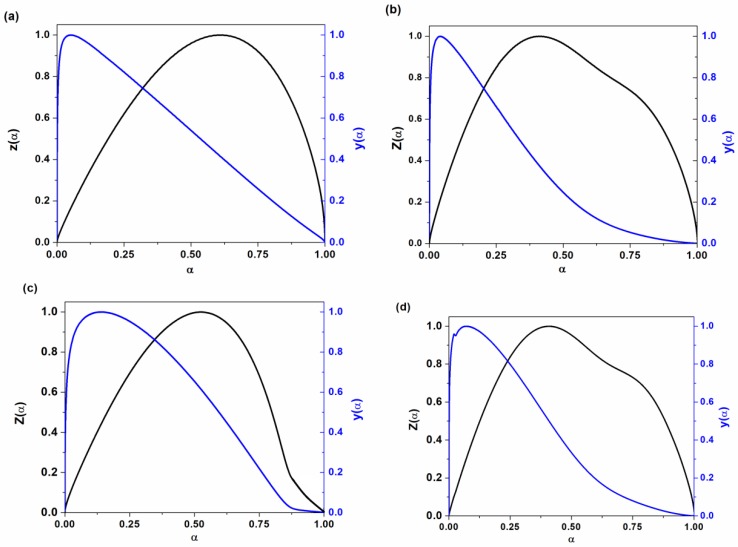
Variation of *y(α)* and *Z(α)* versus conversion for (**a**) neat epoxy, (**b**) epoxy/P-HNT, (**c**) epoxy/A-HNT and (**d**) epoxy/F-HNT nanocomposites based on Malek model.

The experimental values of *y(α)* and *z(α)* for neat epoxy and its nanocomposites containing 0.2 mass% of P-HNT, A-HNT and F-HNT are shown in Figure 9 and compared with theoretical master plots. The curing reaction model for neat epoxy and its nanocomposites can be determined as the best match between the experimental (Figure 9) and theoretical master plots [48]. 

As can be observed from Figure 9, *y(α)* and *z(α)* shows a maximum point at α_m_, and α_p_^∞^, respectively. The values of α_m_, α_p_^∞^ and α_p_ (α_p_ can be obtained from DSC peak) for neat epoxy and its nanocomposites at different heating rates are reported in Table 1. The shapes of y(α_p_) and z(α_p_) in Figure 9 and the values of α_m_ which are lower than α_p_ and α_p_^∞^ < 0.632 indicated two-parameter autocatalytic kinetic model. 

#### 3.2.2. Determining Degree of Reaction

According to the *Friedman* and *Malek* methods the reaction model of neat epoxy and epoxy containing P-HNT, A-HNT and F-HNT correlated with Sestak and Berggren empirical kinetic model. By substituting Equation (9) in Equation (4) the curing reaction rate rewrite as follows:

(14) $$\frac{d\alpha }{dt}=Aexp(-\frac{E_{\alpha }}{RT})\alpha^{m}{\left(1-\alpha \right)}^{n},$$

The kinetic parameters including the degrees of autocatalytic reaction (*n* and *m*) and the frequency factor (*A*) can be determined through the following equations:

(15) $$ValueΙ=ln\left(\frac{d\alpha }{dt}\right)+\frac{E_{\alpha }}{RT}-ln\left[\frac{d(1-\alpha )}{dt}\right]-\frac{E_{\alpha }}{RT^{\prime }}=(n-m)ln\left(\frac{1-\alpha }{\alpha }\right),$$

(16) $$ValueIΙ=ln\left(\frac{d\alpha }{dt}\right)+\frac{E_{\alpha }}{RT}+ln\left[\frac{d(1-\alpha )}{dt}\right]+\frac{E_{\alpha }}{RT^{\prime }}=(n+m)ln(\alpha -\alpha^{2})+2lnA$$

**Figure 10:**
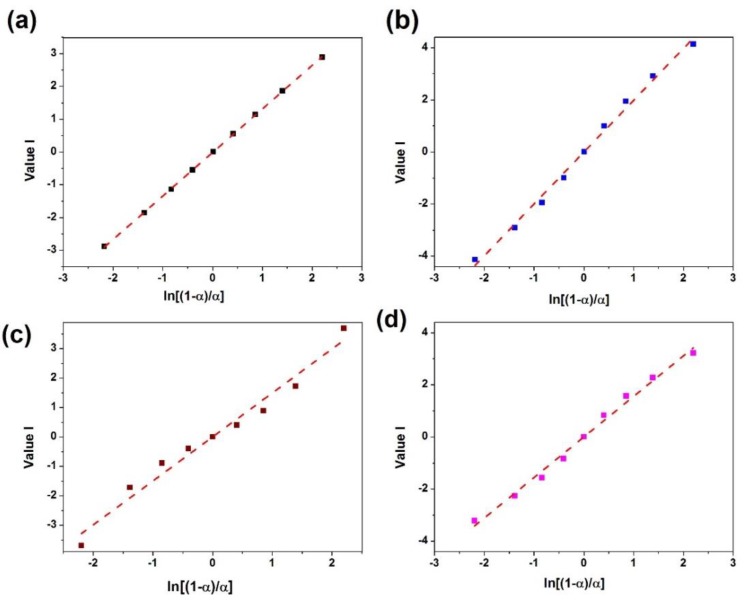
Plots of *ValueI* calculated using DSC data for (**a**) neat epoxy, (**b**) epoxy/P-HNT, (**c**) epoxy/A-HNT and (**d**) epoxy/F-HNT.

**Figure 11:**
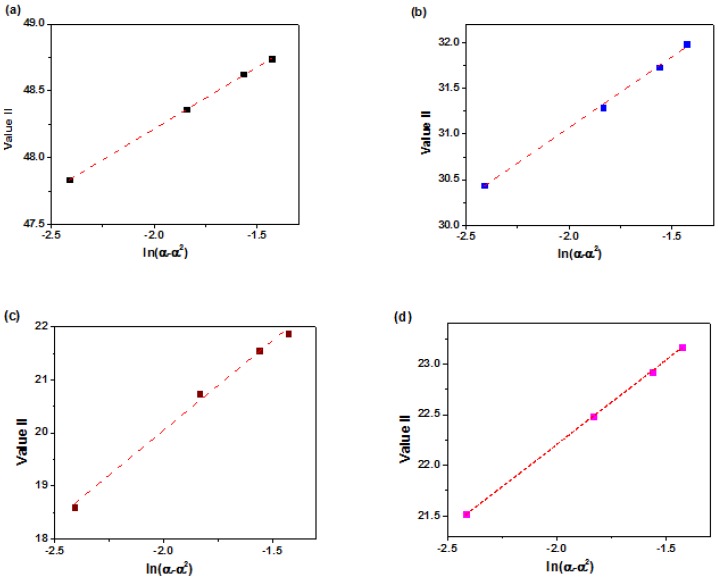
Plots of *ValueII* calculated using DSC data for (**a**) neat epoxy, (**b**) epoxy/P-HNT, (**c**) epoxy/A-HNT and (**d**) epoxy/F-HNT.

By plloting *ValueI* vs. *ln [(1 − α)/α]* a straight line is obtained which slope gives the value of *n − m* (Figure 10). In addition, the value of *n + m* and *2lnA* can be obtained from the slope and intercept of the plot of *ValueII* vs. *ln(α − α^2^)* (Figure 11).

For calculating the values of *m*, *n* and *lnA* from equations of 15 and 16 the *E_α_* value was used as the average amount in different conversion based on *Friedman* and *KAS* methods and reported in Table 2.

As can be observed from Table 2, the trend of variation of kinetic parameters (*n*, *m* and *lnA*) of epoxy system by addition of P-HNT, A-HNT and F-HNT obtained from *Friedman* and *KAS* methods are in well agreement with each other. For neat epoxy and its nanocomposites in all heating rates the overall reaction order (*m + n*) is higher than unity which is indicative of complexity of the curing mechanism [49]. The results of kinetic parameters indicated an increase in autocatalytic reaction order (*m*) by introduction of P-HNT into epoxy matrix. The reaction of hydroxyl groups of P-HNT with epoxy resin push the balance to the benefit of -OH groups on the surface of P-HNT towards epoxide ring opening via etherification autocatalytic reaction [50,51]. In the case of epoxy/A-HNT nanocomposite autocatalytic reaction order is lower in comparison with P-HNT due to the fact that A-HNT has lower hydroxyl groups compared to P-HNT. Because by alkali activation of HNT results inner hydroxyl groups removes as Al(OH)_3_ sheets which decreased the reaction between OH groups and epoxy rings. Moreover, addition of P-HNT, A-HNT and F-HNT decreased collisions between the curing moieties, as reflected in a drop in pre-exponential factor. Moreover, the lower amount of activation energy in the presence of F-HNT also reflects in the lower pre-exponential factor as can be observed in Table 2. This reduction in the frequency factor, which is originated from the number of collisions between curing moieties, is attributed to the reduction of the segmental diffusion rates. 

#### 3.2.3. Model Validation

**Figure 12:**
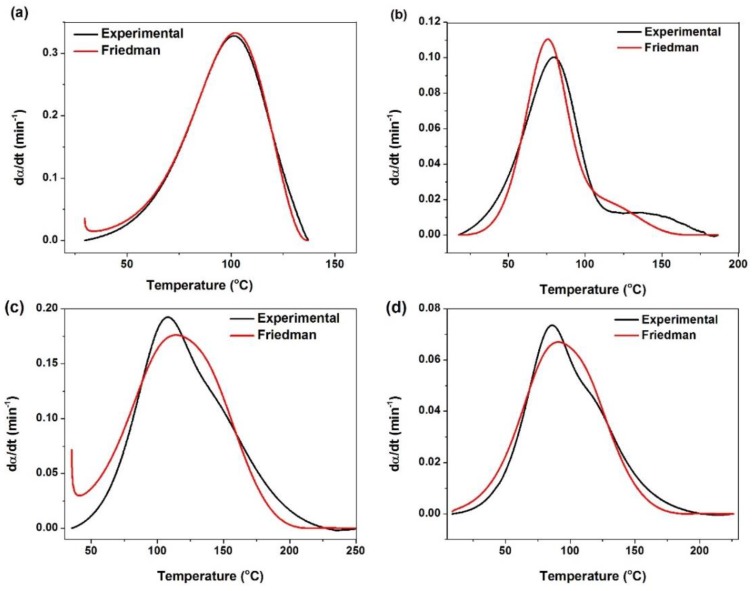
Comparison of experimental data with the kinetic models for (**a**) neat epoxy, (**b**) epoxy/P-HNT, (**c**) epoxy/ A-HNT and (**d**) epoxy/ F-HNT based on *Friedman* model.

**Figure 13:**
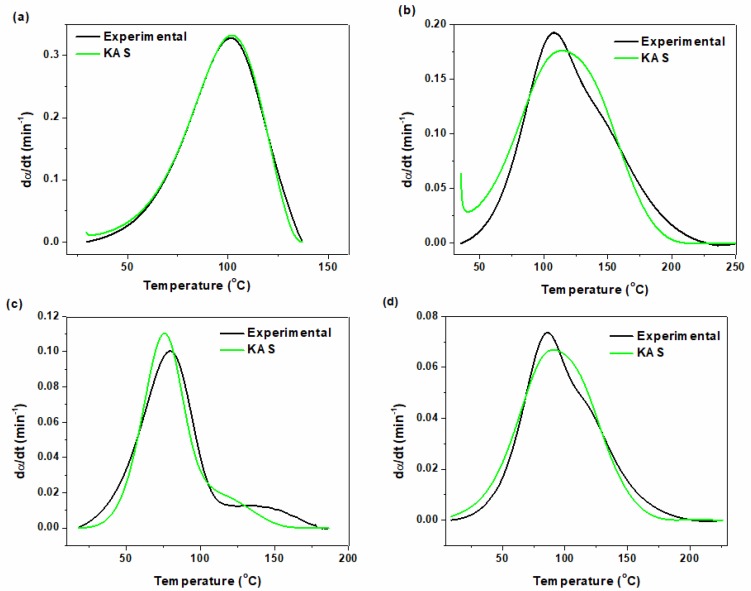
Comparison of experimental data with the kinetic models for (**a**) neat epoxy, (**b**) epoxy/P-HNT, (**c**) epoxy/ A-HNT and (**d**) epoxy/ F-HNT based on *KAS* model.

By estimating activation energy and kinetic parameters, the value of curing rate of epoxy systems can be calculated from Equation (14) and compared with experimental data. Figure 12 and Figure 13 represent the calculated curing rate for neat epoxy and its nanocomposites based on *Friedman* and *KAS* models, respectively, in comparison with the experimental data. As apparent, both *Friedman* and *KAS* approaches match well with each other. In the case of neat epoxy both methods coincide with experimental curve. By contrast, some differences can be seen between experimental and predicted curing for epoxy system in the presence of P-HNT, A-HNT and F-HNT. By introduction of nanotubes the mobility of curing moieties decreased which results in some deviation between the predicted values and the experimental data.

## 4. Conclusions

Nonisothermal DSC was applied to study the cure kinetics of of epoxy/amine systems containing 0.2 wt.% P-HNT, A-HNT and F-HNT. The evolution of activation energy of neat epoxy as a function of curing conversion indicated an increasing trend due to viscosity rise which hindered the mobility of curing moieties. KAS model showed that average value of activation energy decreased from 71 kJ/mol for neat epoxy to about 56, 39 and 46 kJ/mol for epoxy nanocomposites containing P-HNT, A-HNT and F-HNT, respectively. The higher decline in activation energy of epoxy containing F-HNT compared to P-HNT incorporated epoxy system is due to epoxide groups on the surface of HNT which may results in better dispersion state and catalyzing the curing reactions between epoxy resin and curing agent by etherification reaction. By contrast, it was observed that the activation energy of the curing reaction calculated by *Friedman* and *KAS* methods decreased in the presence of A-HNT due to the decrease of OH groups caused by the removal of Al(OH)_3_ sheets from inner surface of HNT. Detailed analysis of cure in terms of kinetics parameters can be performed by monitoring the autocatalytic reaction degree. At low heating rate of 5 °C min^−1^ s, the value of *m* increased from 0.107 for neat epoxy/amine system to the values of 0.908 and 0.24 for the epoxy/P-HNT and epoxy/A-HNT nanocomposites, respectively, whereas epoxy/F-HNT system had *m* value of 0.072 as a sign of dominance of the non-catalytic reactions. This obviously denotes the catalytic effect of A-HNT, and more remarkably P-HNT. At high heating rate of 5 °C min^−1^ s, a similar behavior was observed. It can be concluded that introduction of HNT suppresses the mobility of curing moieties. As a result, deviation of experimental cure rate values from the predicted values obtained by *KAS* and *Friedman* methods was slightly observed. 

## Figures and Tables

**Table 1:** The values of α_p_, α_m_ and α_p_^∞^ obtained from DSC analysis based on *Malek* model at various heating rates.

Designation	Heating Rate (°C/min)	α_p_^∞^	α_m_	α_p_
**Epoxy**	5	0.465	0.055	0.507
	10	0.868	0.023	0.596
	15	0.964	0.052	0.614
	20	0.886	0.040	0.593
**Epoxy/P-HNT**	5	0.370	0.029	0.419
	10	0.363	0.025	0.426
	15	0.347	0.039	0.415
	20	0.288	0.037	0.410
**Epoxy/A-HNT**	5	0.482	0.138	0.528
	10	0.837	0.179	0.616
	15	0.823	0.161	0.614
	20	0.611	0.174	0.546
**Epoxy/F-HNT**	5	0.380	0.070	0.409
	10	0.318	0.052	0.400
	15	0.306	0.042	0.403
	20	0.329	0.046	0.399

**Table 2:** The kinetic parameters evaluated for the curing of pristine epoxy resin and its nanocomposites based on *Friedman* and *KAS* models at different heating rates.

Designation	Heating Rate (°C/min)	*Ē_α_* (kJ/mol)	*ln(A)* (1/s)	Mean (1/s)	*m*	Mean	*n*	Mean
Friedman								
Epoxy	5	78.40	25.08	25.00	0.103	0.158	1.849	1.400
	10		25.18		0.173		1.309	
	15		24.94		0.186		1.133	
	20		24.79		0.170		1.307	
Epoxy/P-HNT	5	57.72	17.02	17.08	0.908	0.511	1.763	1.788
	10		17.02		0.360		1.741	
	15		17.14		0.357		1.800	
	20		17.14		0.419		1.849	
Epoxy/A-HNT	5	39.17	13.40	12.74	0.240	0.213	2.407	1.401
	10		12.49		0.246		0.943	
	15		12.56		0.186		0.978	
	20		12.49		0.181		1.277	
Epoxy/F-HNT	5	43.69	12.76	12.69	0.072	0.056	1.620	1.626
	10		12.78		0.031		1.683	
	15		12.63		0.045		1.623	
	20		12.59		0.077		1.578	
KAS								
Epoxy	5	71.15	22.66	22.66	0.013	0.060	1.767	1.343
	10		22.82		0.070		1.260	
	15		22.63		0.086		1.090	
	20		22.51		0.070		1.257	
Epoxy/P-HNT	5	56.50	16.62	16.70	0.911	0.515	1.743	1.768
	10		16.64		0.365		1.721	
	15		16.76		0.362		1.780	
	20		16.77		0.423		1.828	
Epoxy/A-HNT	5	38.84	13.29	12.63	0.220	0.195	2.401	1.398
	10		12.39		0.227		0.941	
	15		12.45		0.169		0.976	
	20		12.38		0.164		1.274	
Epoxy/F-HNT	5	46.27	13.61	13.50	0.036	0.062	1.664	1.671
	10		13.60		0.007		1.729	
	15		13.42		0.085		1.668	
	20		13.37		0.118		1.623	
